# Photoactivated Platinum Complexes: Targeting Membrane Proteins and Organelles for Precision Cancer Therapy

**DOI:** 10.1002/anie.5354099

**Published:** 2026-08-27

**Authors:** Shu Chen, Qiyuan Zhou, Jiaqian Xu, Guangyu Zhu

**Affiliations:** ^1^ Department of Chemistry City University of Hong Kong Hong Kong SAR China; ^2^ City University of Hong Kong Shenzhen Research Institute Shenzhen China

**Keywords:** Cancer cell targeting, organelle targeting, photoactivated chemotherapy, photodynamic therapy, platinum anti‐tumor agents, precision therapy

## Abstract

Photodynamic therapy (PDT) and photoactivated chemotherapy (PACT) have emerged as promising anticancer modalities due to their noninvasive nature, spatiotemporal precision, and high tumor suppression efficacy. Integrating platinum with these phototherapies addresses key limitations of conventional platinum chemotherapy, specifically systemic toxicity and pervasive drug resistance. Since cancer cell membrane proteins, including receptors and transporters, and subcellular organelles play a pivotal role as therapeutic targets, directing platinum phototherapeutics to membrane proteins or specific organelles markedly enhances efficacy compared to nonspecific targeting. Consequently, membrane protein‐ and organelle‐targeted platinum‐based phototherapeutics represent a transformative advance in cancer treatment. This review highlights recent advancements in membrane protein‐/organelle‐targeted and photoactivated platinum complexes and explains how targeting improves efficacy, reduces resistance, and has the potential to minimize off‐target toxicity. We examine the therapeutic benefits derived from localized generation of reactive oxygen species, light‐triggered release of cytotoxic species, and activation of organelle‐specific cell death pathways. These insights may guide the rational design of next‐generation photoactivatable platinum complexes, offering opportunities for improved therapeutic outcomes through synergistic mechanisms and precise targeting strategies.

## Graphical abstract

This review highlights an emerging anticancer approach integrating platinum agents into photodynamic therapy (PDT) and photoactivated chemotherapy (PACT) systems, further augmented by cancer cell membrane‐ and organelle‐targeting. By directing photoactivated platinum complexes from nontargeted distribution to cancer cells or defined subcellular sites, these systems aim to increase local drug activation and damage, thereby amplifying therapeutic efficacy, overcoming drug resistance, and reducing off‐target effects.

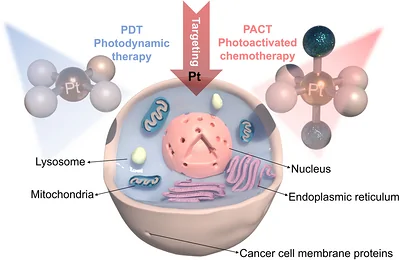

## 1 Introduction

Photodynamic therapy (PDT) is a clinically approved, minimally invasive treatment that combines a photosensitizer (PS), molecular oxygen (O_2_), and light to generate cytotoxic reactive oxygen species (ROS) for targeted tumor ablation [1, 2, 3]. As illustrated in Figure 1a, the PDT process begins with the photoexcitation of the PS from its ground singlet state (S_0_) to an excited singlet state (S_1_), followed by intersystem crossing (ISC) to a metastable triplet state (T_1_) [4, 5]. This triplet‐state PS then triggers two possible photochemical pathways: a Type I mechanism, which involves electron transfer with biomolecules to generate ROS (such as superoxide and hydroxyl radicals), and a Type II mechanism, which transfers energy to O_2_ to produce singlet oxygen (^1^O_2_) [2, 6, 7]. Most PSs predominantly follow the oxygen‐dependent Type II mechanism, while Type I pathways are generally less common and can be efficiently activated in hypoxic conditions [8, 9]. PDT exploits the short diffusion radius (approximately 100 nm) of ROS to induce localized oxidative damage at illuminated tumor sites, achieving high spatiotemporal precision, minimizing systemic toxicity and adjacent tissue damage, while also circumventing drug resistance, making it a promising alternative to conventional cancer therapies [2, 10, 11, 12]. Despite this precision, PDT's oxygen‐dependent Type II ROS generation is limited in hypoxic tumor microenvironments (TMEs), motivating oxygen‐independent alternatives such as photoactivated chemotherapy (PACT) [13, 14]. PACT utilizes light to activate inert compounds, which release cytotoxic species without relying on molecular oxygen [15]. Operating through a mechanism similar to Type I PDT (Figure 1a), PACT agents in their excited states undergo electron transfer, leading to prodrug degradation and the release of cytotoxic entities such as parent drugs and radicals that induce cancer cell death [16, 17]. Additionally, the controlled activation of inert prodrugs via PACT minimizes off‐target toxicity, enhancing selectivity and expanding their potential for precise anticancer applications [18].

**FIGURE 1:**
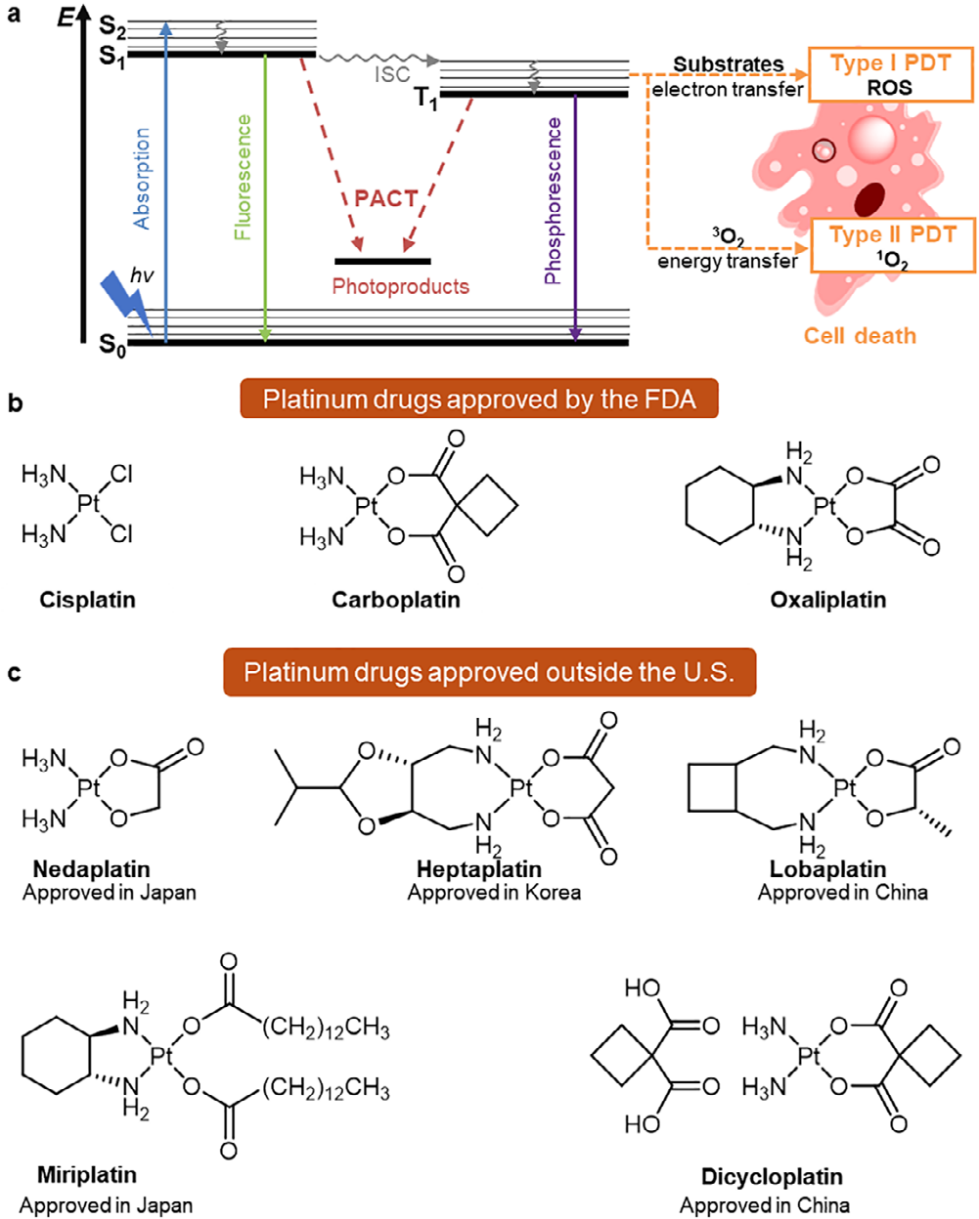
(a) Jablonski diagram depicting Type I and Type II PDT, as well as PACT. (b,c) The chemical structures of Pt‐based anticancer drugs that have been approved for clinical applications.

Platinum (Pt)‐based chemotherapeutic agents, including cisplatin, carboplatin, and oxaliplatin (Figure 1b), have revolutionized cancer treatment and markedly improved prognoses since their introduction; subsequent generations, such as region‐specific derivatives (Figure 1c), have further broadened their clinical applications [19, 20, 21, 22]. The discovery of cisplatin's anticancer properties also prompted the development of numerous Pt(II) and Pt(IV) derivatives, advancing research into agents with superior efficacy [22, 23]. Traditional Pt(II) drugs exert their cytotoxic effects through intracellular aquation, which facilitates DNA crosslinking that subsequently induces apoptosis [24, 25]. However, the efficacy of Pt drugs is often hampered by a lack of tumor selectivity, systemic toxicity, and both inherent and acquired resistance [26]. These challenges necessitate innovative strategies to enhance tumor targeting, mitigate toxicity, and circumvent resistance, ultimately extending the clinical potential of Pt‐based therapies.

Integrating Pt agents with PDT and PACT creates dual phototherapeutic modalities that address the limitations of conventional Pt‐based treatments via light‐activated mechanisms [27, 28]. In PDT, Pt‐coordinated PSs leverage the heavy metal effect to enhance intersystem crossing, boosting ROS generation and anticancer efficacy upon irradiation [1, 6]. By contrast, Pt‐based PACT utilizes relatively inert Pt(IV) complexes that undergo oxygen‐independent, spatiotemporally controlled photoactivation to release cytotoxic Pt(II) species—either alone, sometimes together with bioactive ligands, or alongside azidyl radicals and ROS [29, 30, 31]. Despite the precise activation afforded by light irradiation, conventional photoactivatable Pt complexes lack intrinsic cancer cell specificity. This nonselective biodistribution compromises tumor accumulation and elevates the risk of phototoxicity to adjacent healthy tissue [32, 33]. Consequently, various targeting strategies have been developed to direct photoactivatable Pt complexes selectively to malignant cells, with the aim of augmenting both the safety and efficacy of PDT and PACT [34]. Furthermore, given that photoactivation occurs at the target site, the resulting cytotoxicity is largely confined to diseased tissue, minimizing collateral damage and reducing side effects. Both PDT (ROS‐mediated) and PACT (cytotoxic species‐mediated) exhibit spatiotemporal selectivity due to the restricted diffusion of their active agents [1, 15]. For example, ROS have ultrashort lifespans (ranging from 0.03 to 0.18 ms in biological systems) and limited diffusion ranges; similarly, PACT cytotoxic species typically act proximally to their generation site [35, 36]. Consequently, the therapeutic effectiveness of both approaches hinges critically on the subcellular localization of PSs or photoactivated agents. Targeted delivery to vital or vulnerable organelles concentrates ROS or cytotoxic species, causing direct damage to critical components and enhancing efficacy compared to nonspecific distribution [37, 38]. Key organelles—including the nucleus, mitochondria, lysosomes, and endoplasmic reticulum (ER)—are therefore compelling therapeutic targets [39, 40]. Disrupting or destroying these organelles compromises cellular integrity and triggers cell death. Following this rationale, deliberate localization of PSs or photo‐activated agents to these organelles enhances therapeutic precision by offering two principal advantages: (1) localized accumulation maximizes ROS or cytotoxic species generation at lethal sites, surpassing diffusion limitations and improving damage efficiency [41], and (2) confinement within organelles minimizes agent efflux, decreasing required dosages and mitigating the risk of multidrug resistance (MDR) [42].

This review systematically: (i) outlines recent progress in cancer cell membrane protein‐ and organelle‐targeted Pt‐based phototherapeutics (Figure 2), (ii) elucidates the mechanisms underlying their enhanced cytotoxicity, ability to overcome resistance, and reduced off‐target effects, and (iii) delineates future research directions, positioning these complexes as promising candidates for precise cancer therapy. To maintain a focus on small molecules, this discussion excludes Pt‐based and targeted nanostructured materials, which deliver Pt to various organelles and have been extensively reviewed elsewhere [43, 44].

**FIGURE 2:**
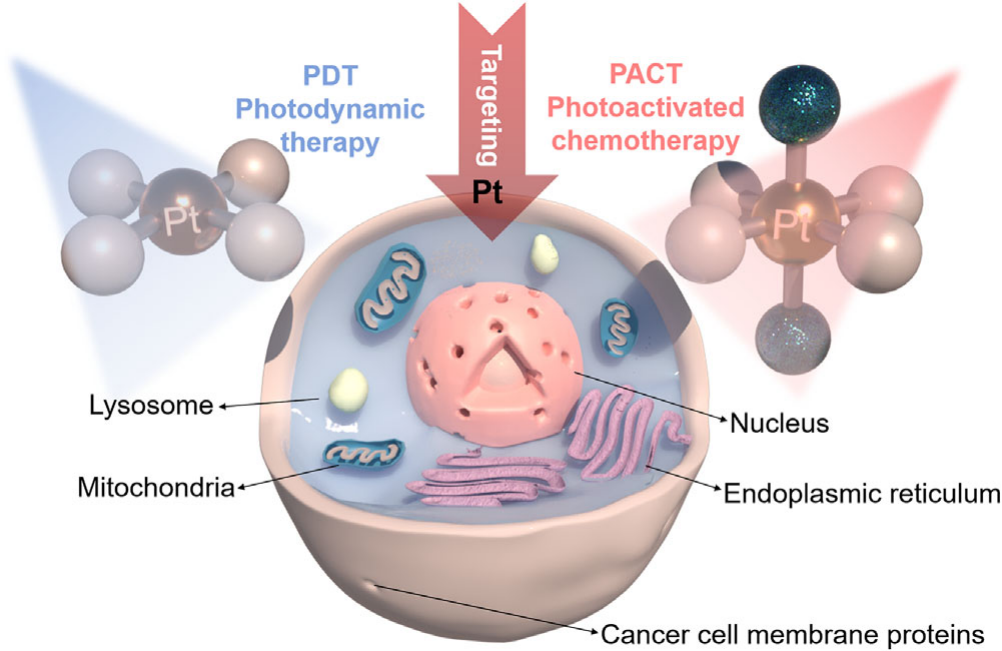
Illustration of cancer cell membrane protein‐ and organelle‐targeting phototherapeutic Pt complexes for precision cancer therapy.

## 2 Cancer Cell Membrane Protein‐Targeting Photoactivatable Pt Complexes

In cancer cells, dysregulated proliferation is often associated with the overexpression of specific membrane proteins, including folate receptors, integrins, and glucose transporters [45, 46]. The substantial differences in the expression of these surface markers between tumor and normal cells provide a strong basis for active targeting strategies [47]. Accordingly, photoactivatable Pt complexes can be functionalized with specific ligands to recognize and bind these overexpressed receptors or transporters, thereby promoting selective cellular uptake and efficient intracellular transport. These complexes exploit tumor‐associated membrane proteins exclusively for targeted recognition and receptor‐mediated internalization, rather than for permanent membrane anchoring or direct membrane damage. This targeted accumulation can enhance cancer cell selectivity by increasing drug enrichment in malignant cells relative to normal tissues. Upon photoactivation, the internalized Pt complexes can then induce cellular damage, ultimately leading to augmented anticancer activity.

### 2.1 Targeting Folate Receptors

Folate receptors (FRs), which are overexpressed in cancer cells due to their heightened folate demands for sustaining rapid proliferation, present promising targets for selective drug delivery [46, 48, 49]. Folic acid (FA, compound **1**, Figure 3a) is comprised of three structural components—a pterin ring, aminobenzoate, and glutamate—that facilitate its binding to FRs [50]. The pteridine ring enables strong hydrogen bonding and hydrophobic interactions between its N/O atoms and the residues in the receptor's binding pocket [51]. Crucially, the glutamate moiety extends beyond the binding site, allowing its carboxylate groups to be available for conjugation without compromising FR binding affinity. Through conjugation of phototherapeutic Pt complexes to the glutamate carboxylate group, FA can specifically guide non‐selective Pt drugs into cancer cells, leading to elevated accumulation in tumor cells and improved tumor cell‐selectivity compared to untargeted analogues.

**FIGURE 3:**
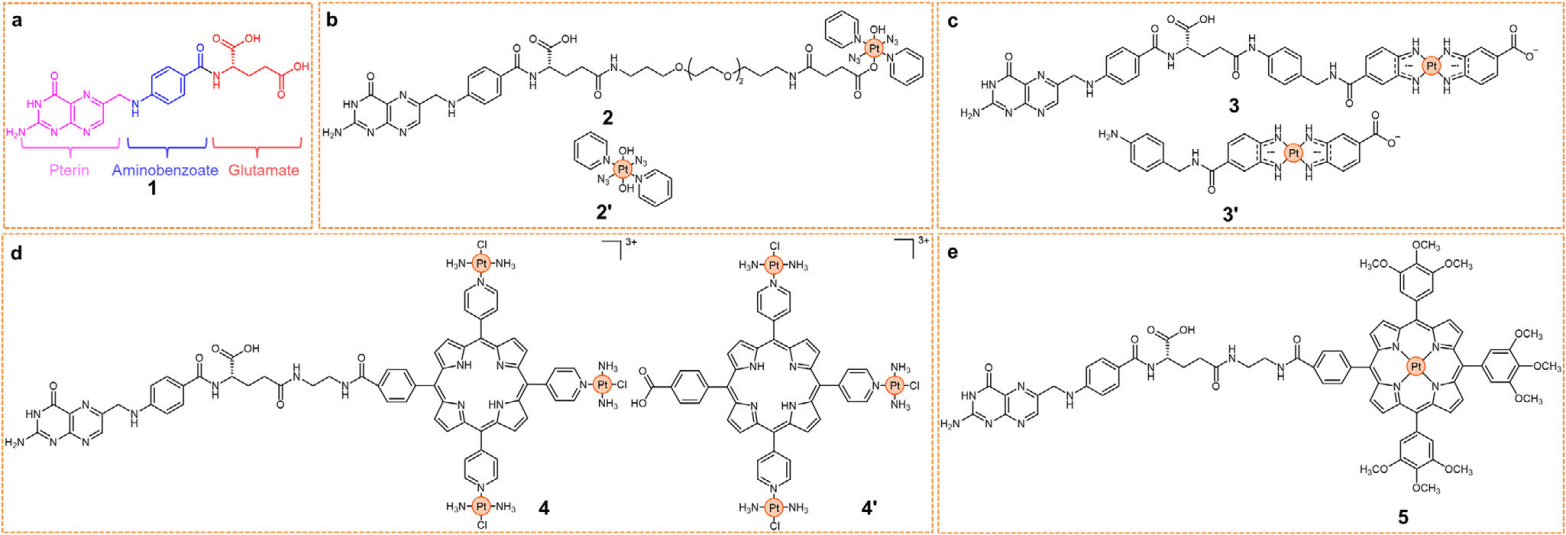
Folate receptor‐targeting photoactivatable Pt anticancer complexes and their non‐targeting analogues.

Marchán, Sadler, and coworkers synthesized an FR‐targeted photoactivatable prodrug by conjugating a photoactivatable *trans*‐diazido Pt(IV) prodrug with FA, affording complex **2** (Figure 3b) [52]. The FA conjugation conferred selective targeting of FR‐overexpressing cancer cells, achieving an 8.6‐fold increase in cellular Pt accumulation relative to its nontargeting analogue, complex **2′**. Upon blue‐light irradiation, complex **2** exhibited significantly superior photocytotoxicity to **2′**, attributable to both increased intracellular Pt accumulation and the generation of a set of different cytotoxic species, including not only photogenerated Pt(II) species but also pterin‐derived ROS. Separately, Sawamura et al. developed FA‐functionalized diradical‐Pt(II) complex **3** (Figure 3c), which exhibited 11.1‐fold higher cellular Pt accumulation in FR‐overexpressing MCF‐7 cells versus its nontargeted analogue **3′**, further exemplifying FA's role in enhancing uptake [53].

Folate conjugation significantly enhances porphyrin tumor targeting and thus amplifies its PDT efficacy [54]. For example, a platinated porphyrin‐FA conjugate (complex **4**, Figure 3d) demonstrated FR‐specific uptake via receptor‐mediated endocytosis and lysosomal localization in FR‐positive cells, unlike its nontargeted analogue (complex **4'**), which failed to internalize [55]. Complex **4** showed potent light‐activated cytotoxicity with minimal dark cytotoxicity in FR‐overexpressing cells. Similarly, the Pt‐based porphyrin‐FA complex **5** (Figure 3e) exhibited selective FR‐mediated uptake and potent FR‐specific phototoxicity upon irradiation, alongside negligible dark cytotoxicity [56]. This enhanced phototoxicity was attributed to the combined effects of increased cellular internalization and amplified singlet oxygen generation driven by the Pt‐mediated heavy atom effect.

### 2.2 Targeting Integrin

Integrins are a family of heterodimeric transmembrane glycoproteins composed of noncovalently linked α and β subunits, and they represent compelling therapeutic targets due to their overexpression in tumors [57, 58]. This receptor family regulates critical processes involved in tumor progression and metastasis, including cell survival, proliferation, and angiogenesis [59]. Among integrins, α_v_β_3_ stands out as a key mediator of tumor growth, invasiveness, and metastasis, while also contributing to pathological angiogenesis and inflammatory disorders [60]. Notably, α_v_β_3_ is highly expressed in tumor cells and activated endothelial cells, in contrast to its minimal presence in quiescent endothelial cells and most healthy tissues [61]. Its high‐affinity recognition of the tripeptide motif arginylglycylaspartic acid (RGD) facilitates the development of targeted delivery systems using RGD‐based peptides for tumor‐specific chemotherapy and imaging applications [62, 63].

Gandioso et al. developed a photoactivatable Pt(IV) prodrug conjugated to a cyclic RGD peptide, yielding complex **6** (Figure 4a) for α_v_β_3_ integrin‐targeted therapy [64]. Upon UVA irradiation (365 nm), complex **6** exhibited 6.3‐fold higher cytotoxicity in α_v_β_3_‐overexpressing SK‐MEL‐28 melanoma cells versus low‐α_v_β_3_ DU‐145 prostate carcinoma cells. This effect correlated with a 2.8‐fold increase in Pt accumulation in SK‐MEL‐28 cells relative to DU‐145 cells and significantly exceeded the accumulation observed with its non‐targeted analogue **6′**, confirming enhanced cellular accumulation via RGD‐mediated targeting. Similarly, Sarli's group synthesized a cyclometalated N^C^N‐Pt(II)‐c(RGDyK) luminophore (complex **7**, Figure 4b), which showed markedly enhanced fluorescence and cellular accumulation via α_v_β_3_‐mediated endocytosis in U87 glioblastoma cells, as well as superior PDT‐induced cytotoxicity compared to its non‐targeting analogue **7′** [65].

**FIGURE 4:**
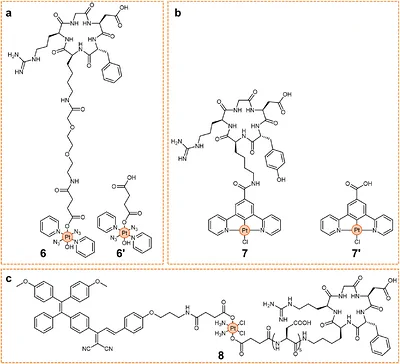
Integrin‐targeting photoactivatable Pt anticancer complexes and their nontargeting analogues.

Beyond enhancing photocytotoxicity, targeting can also overcome drug resistance. For example, Yuan et al. designed a targeted Pt prodrug **8** (Figure 4c) conjugated with an aggregation‐induced emission (AIE) PS to address cisplatin resistance [66]. Complex **8** demonstrated selective and significantly higher accumulation in α_v_β_3_‐overexpressing MDA‐MB‐231 and U87‐MG cells compared to low‐α_v_β_3_ MCF‐7 cancer cells and non‐cancerous 293T controls. This receptor‐mediated targeting conferred both selectivity and reduced off‐target toxicity, as evidenced by the prodrug's minimal cytotoxicity in MCF‐7 and 293T cells under both dark and irradiated conditions. Upon irradiation, complex **8** exhibited a ninefold increase in potency against cisplatin‐resistant MDA‐MB‐231 cells (IC_50_ = 4.2 vs. 37.1 µM in darkness), effectively bypassing chemoresistance through targeted delivery and photodynamic activation, in contrast to cisplatin's static IC_50_ of 33.4 µM, which is light‐independent.

### 2.3 Targeting Glucose Transporter 1

To maintain proliferative homeostasis, cancer cells reprogram their glucose metabolism to enhance glycolysis, a phenomenon known as the Warburg effect, which is driven by mitochondrial metabolic alterations [67]. This metabolic reprogramming necessitates increased glucose uptake via the overexpression of membrane transporters, particularly glucose transporter 1 (GLUT1). GLUT1 is ubiquitously upregulated across diverse malignancies, including breast, lung, colorectal, and pancreatic cancers [68]. This cancer‐specific reliance positions GLUT1 as a strategic target for tumor‐selective therapies [69]. Recent advances leverage glucose moieties conjugated to therapeutic or diagnostic agents, exploiting GLUT1 overexpression to enhance tumor‐specific delivery. This approach not only improves targeting precision but also augments the aqueous solubility of metal‐based complexes, amplifying cellular accumulation and therapeutic efficacy.

Chakravarty, Kondaiah, and coworkers demonstrated that the glucose‐functionalized Pt(II)‐BODIPY complex **9** (Figure 5a) acted as a targeted PDT agent under red light irradiation, exhibiting significantly higher cytotoxicity in malignant cells than its non‐glucose analogue **9′** [70]. This enhanced efficacy stemmed from glucose‐mediated selective cellular accumulation, as evidenced by a 3.2‐fold increase in intracellular Pt levels in the A549 cancer cells relative to **9′**. Furthermore, the significantly lower accumulation and non‐toxic profile of complex **9** in normal HPL1D cells underscored the role of the glucose moiety in achieving selective cancer targeting while minimizing toxicity in normal cells. Building on this targeting strategy, a glucose‐conjugated curcumin‐Pt(II) complex (**10**, Figure 5b) was subsequently developed [71]. Complex **10** induced photoinduced apoptosis via ROS generation under 400–700 nm light irradiation, showing potent cancer cell photocytotoxicity with minimal dark cytotoxicity, underscoring curcumin's essential role in photocytotoxicity. Glyco‐conjugation of complex **10** enhanced its cellular uptake via GLUT‐mediated transport, as validated by a 10‐fold decrease in accumulation under high glucose competition and significantly greater accumulation compared to its non‐glycosylated control **10′**, thereby yielding a 2.5‐fold increase in phototoxicity relative to **10′**. Accumulation and cytotoxicity were markedly lower in HPL1D normal cells than in A549 cancer cells, indicating pronounced cancer cell‐selectivity (selectivity index, SI = 3.5; defined as IC_50_ in normal cells/IC_50_ in cancer cells)—conferred by GLUT‐targeting. Extending this design, the glucose‐functionalized Pt(II) BODIPY metallacycle (**11**, Figure 5c) exemplified dual‐targeted anticancer agents by combining Pt(II) chemotherapy with BODIPY‐based PDT [72]. Complex **11** exploited GLUT1‐mediated endocytosis for selective cancer cell uptake, corroborated by a twofold increase in intracellular fluorescence in malignant versus noncancerous cells and by glucose‐competition assays. It also exhibited high singlet oxygen quantum yields, resulting in potent and selective photocytotoxicity against cancer cells with negligible dark toxicity. Notably, complex **11** retained significant efficacy against drug‐resistant cells, effectively overcoming chemoresistance and highlighting its therapeutic potential.

**FIGURE 5:**
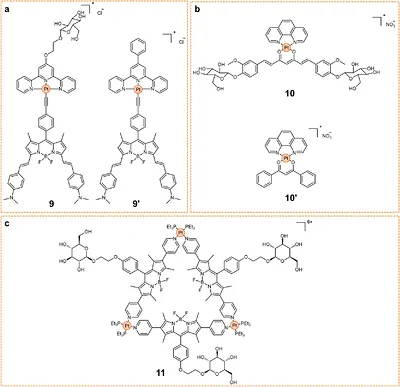
GLUT1‐targeting photoactivatable Pt anticancer complexes and their nontargeting analogues.

## 3 Organelle‐Targeting Photoactivatable Pt Complexes

ROS possess ultrashort half‐lives (0.03–0.18 ms in biological systems) and restricted diffusion distances; likewise, the cytotoxic species generated in PACT act predominantly near their site of production [35, 36]. Consequently, the subcellular localization of PSs or photoactivated agents is a critical determinant of therapeutic efficacy in both PDT and PACT. Subcellular organelles—including the nucleus, mitochondria, lysosomes, and ER—govern core metabolic pathways and maintain the delicate balance between cell proliferation and cell death [34, 73]. Therefore, targeted delivery of photoactivatable platinum complexes to these organelles represents a promising strategy: by concentrating ROS or cytotoxic species at vital intracellular sites, it inflicts direct damage to essential components, thereby outperforming non‐specific distribution and enhancing therapeutic outcomes.

### 3.1 Nucleus‐Targeting Photoactivatable Pt Complexes

The nucleus, which houses chromosomal DNA and regulates essential cellular processes—including proliferation, apoptosis, and metabolism—serves as the central hub for maintaining genomic integrity and cell viability [74, 75]. Pt‐based chemotherapeutics, such as cisplatin, exert their cytotoxic effects primarily through DNA damage; however, their efficacy is limited by inadequate nuclear accumulation, with less than 10% of administered drugs forming DNA adducts [22, 76]. Approximately 85% of these drugs interact nonspecifically with cytoplasmic proteins, which may contribute to systemic toxicity and resistance [77]. The challenge of achieving sufficient nuclear drug delivery is further complicated by the selective permeability of the nuclear membrane, where the lipid bilayer and nuclear pore complexes define the organelle's structure and serve as a barrier to metal complexes, thereby hindering the transport of anticancer agents into the nucleus [78, 79]. Enhancing the nuclear delivery of Pt complexes such as Pt‐based PACT agents could increase DNA damage, overcome resistance mechanisms, and improve therapeutic outcomes [79, 80]. Additionally, ROS exacerbate nuclear vulnerability through lipid peroxidation, DNA strand breaks, and nuclear pore expansion, synergizing with DNA‐damaging agents to induce apoptosis [81, 82, 83]. Therefore, integrating nuclear‐targeting strategies with ROS‐generating mechanisms in Pt drug design presents a dual approach to enhance therapeutic efficacy, reduce off‐target effects, and address resistance challenges in conventional cancer therapies.

Nuclear localization sequences (NLS), which are short peptide tags recognized by nuclear importins for nuclear translocation [84, 85], are increasingly being conjugated to cytotoxic agents like Pt prodrugs for precise nuclear targeting. For example, the NLS (R_8_K, a cell‐penetrating peptide)‐functionalized coumarin‐photocaged Pt(IV) prodrug coumaplatin (**12**, Figure 6a) achieved significantly enhanced nuclear accumulation (68% vs. 12%) and a 6‐ to >100‐fold increase in phototherapeutic efficiency over its nontargeted **12′** [86]. Complex **12** also showed improved cancer cell selectivity relative to complex **12′**, with an SI of 1.9 and 1.0, respectively. Upon photoactivation, complex **12** outperformed oxaliplatin in DNA binding efficiency (more than a twofold increase) and phototoxicity (up to 96‐fold enhancement). It also effectively circumvented Pt resistance: whereas oxaliplatin showed resistance factors (RFs, defined as the IC_50_ in resistant cells/the IC_50_ in sensitive cells) of 5.1 and 2.8 in Pt‐resistant A2780cisR and A549cisR cells, respectively, photoactivated complex **12** displayed markedly reduced RFs of just 1.3 and 0.6. Notably, the nuclear localization of complex **12** facilitated a unique mechanism of action distinct from conventional Pt drugs in Pt‐resistant cells. It induced p53‐independent senescence, immunogenic cell death (ICD) with T cell activation, and light‐triggered cytotoxicity—features absent in oxaliplatin.

**FIGURE 6:**
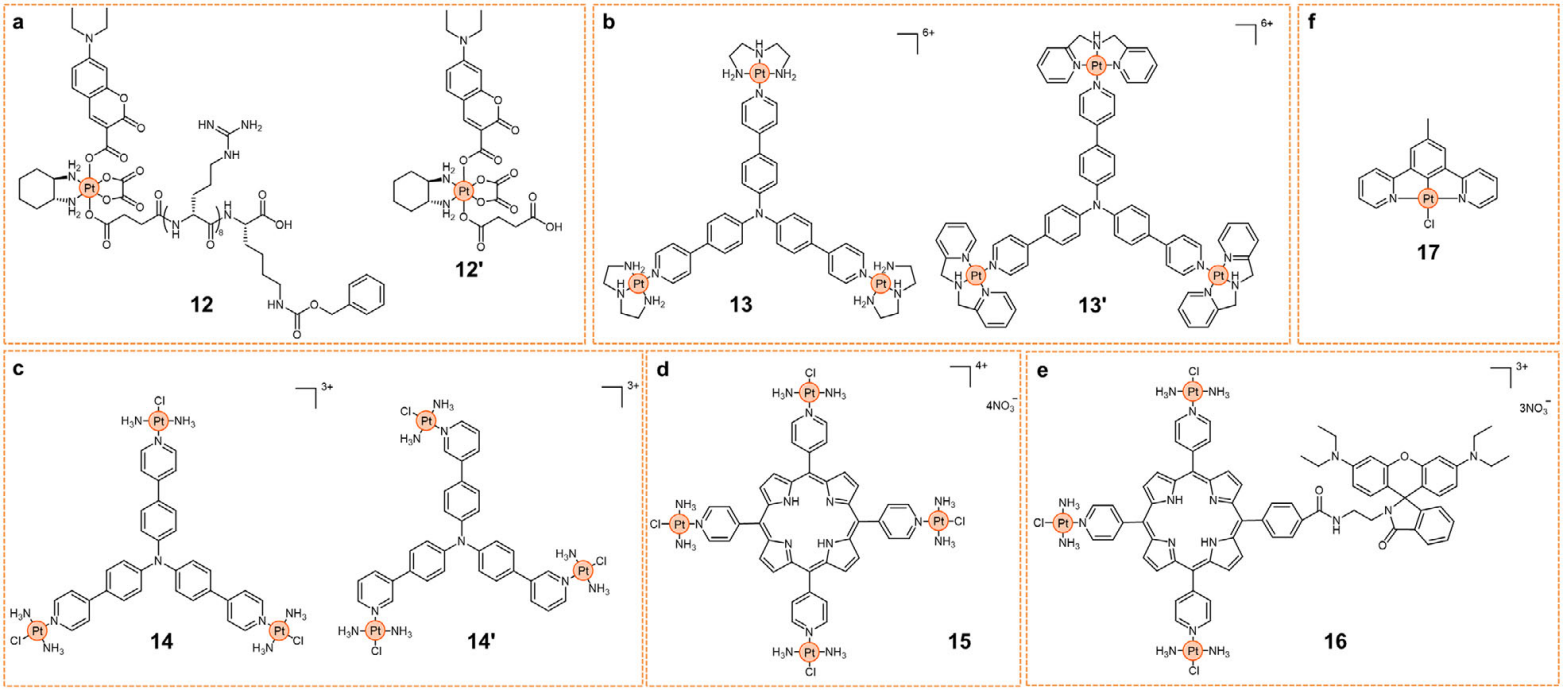
Nucleus‐targeting photoactivatable Pt anticancer complexes and their nontargeting analogues.

Beyond NLS, triphenylamine derivatives have emerged as potent nuclear‐targeting motifs for precision PDT. Mao's group developed triphenylamine‐Pt(II) complex **13** (Figure 6b) [87], which exhibited significantly high phototoxicity in A549 cells, a 26‐fold increase compared to its cytoplasmic‐localizing analogue **13′**. Moreover, complex **13** retained robust efficacy in cisplatin‐resistant A549cisR cells, highlighting its ability to circumvent resistance. Notably, complex **13** exhibited a favorable selectivity against cancerous cells, as indicated by an SI of 4.0. The superior photocytotoxicity of complex **13** stemmed from its preferential nuclear localization and enhanced ROS generation. Building on this strategy, a refined para‐substituted triphenylamine‐Pt(II) complex **14** (Figure 6c) demonstrated superior PDT efficacy—with a 9‐ to 58‐fold enhancement over its meta‐substituted analogue **14′**—attributed to its preferential nuclear (vs. cytoplasmic for complex **14′**) localization and higher singlet oxygen quantum yield [88]. Notably, complex **14** achieved a greater potency in cisplatin‐resistant A549cisR cells than in cisplatin‐sensitive A549 cells, with an RF of 0.4, effectively overcoming resistance. Moreover, complex **14** exhibited lower phototoxicity in noncancerous LO2 cells than in cancerous cells, demonstrating greater selectivity (SI = 2.4). Additionally, complex **14** induced light‐triggered DNA damage, G_2_/M arrest, and apoptosis, with its potent anti‐tumor activity confirmed in xenograft models.

Platinum conjugation to porphyrin boosts singlet oxygen quantum yield and therapeutic outcomes via the heavy‐atom effect [89]. Exemplifying this, Naik et al. synthesized a tetraplatinated porphyrin **15** (Figure 6d), which selectively accumulated in the nuclei, achieving 30‐fold higher Pt levels in HeLa nuclei compared to cisplatin [90]. While displaying negligible dark toxicity, complex **15** exhibited exceptional photocytotoxicity, with a 1200‐fold increase in photocytotoxicity in HeLa cells. Moreover, complex **15** effectively overcame cisplatin resistance, demonstrating potent photocytotoxicity in both cisplatin‐sensitive A2780 and cisplatin‐resistant CP70 human ovarian cancer cells, achieving an RF value of 0.9. Mechanistic investigations revealed intercalative binding to ctDNA and light‐induced DNA cleavage, identifying DNA as a likely primary target. Inspired by Pt‐porphyrin conjugation, Wang, Wong, Wu, and coworkers developed a dual‐targeted platinated porphyrin (**16**, Figure 6e) that localized to both the cytoplasm and nuclei [91]. The critical advantage of nuclear targeting was that it directly facilitated Pt‐DNA adduct formation following photoactivation and efficiently generated singlet oxygen within the nucleus. This dual action yielded potent photocytotoxicity without dark toxicity, allowing for highly precise PDT. Additionally, Doherty et al. reported a charge‐neutral, photostable Pt(II) complex **17** (Figure 6f), which demonstrated potent light‐dependent cytotoxicity and successfully overcame resistance in cisplatin‐resistant cells [92]. Nuclear‐localized **17** induced only transient DNA damage in dark conditions; however, photoactivation caused extensive and persistent DNA damage, ultimately leading to cell death.

### 3.2 Mitochondria‐Targeting Photoactivatable Pt Complexes

Mitochondria are double‐membrane organelles that produce over 80% of ATP in eukaryotic cells and serve as central hubs for energy metabolism, metal homeostasis, apoptosis regulation, and oxidative stress management [93, 94, 95]. Dysfunction in mitochondria disrupts cellular homeostasis and directly activates cell death pathways, particularly through photodamage‐induced collapse of membrane potential and subsequent apoptosis [96, 97]. Unlike nuclear DNA (nDNA), mitochondrial DNA (mtDNA) lacks histone protection and key repair pathways such as nucleotide excision repair (NER), rendering it highly vulnerable to ROS, carcinogens, and radiation [98]. The absence of NER, combined with mtDNA's inherently high mutation rate, further increases its susceptibility to Pt‐based drugs [99, 100]. These unique characteristics highlight mitochondria's potential as valuable therapeutic targets in anticancer strategies. Mitochondria‐targeting approaches leverage the organelle's electronegative outer membrane by employing lipophilic cationic complexes or agents modified with functional groups or peptides [101, 102]. As a result, mitochondria are considered critical targets for Pt‐based photoactivated therapies, which induce controlled mitochondrial disruption to deplete cellular energy reserves and trigger apoptosis.

BODIPY, a well‐established mitochondria‐targeting fluorophore, serves as the functional unit to deliver Pt complexes to mitochondria for enhanced therapeutic outcomes [103]. For example, Xie, Chen, and coworkers synthesized an NIR fluorescent BODIPY‐Pt conjugate (complex **18**, Figure 7a) integrating NIR imaging, chemotherapy, and PDT [104]. Complex **18** accumulated in mitochondria in HepG2 cells, showing superior phototoxicity over cisplatin under 620 nm irradiation, with negligible dark cytotoxicity. Additionally, it enabled real‐time in vivo fluorescence imaging and demonstrated potent therapeutic efficiency in U14 tumor‐bearing mice. Similarly, a pyriplatin‐BODIPY conjugate (**19**, Figure 7b) showed significant mitochondrial colocalization and light‐activated singlet oxygen generation, resulting in potent photocytotoxicity that outperformed the non‐targeting complex **19′** by a factor of 44 to over 769 across multiple cell lines [105]. Building on this approach, a Pt(II)‐BODIPY complex (**20**, Figure 7c) efficiently generated ROS, achieving a 70–100‐fold increase in cytotoxicity against A549 lung cancer cells and multidrug‐resistant MDA‐MB‐231 breast cancer cells compared to the free BODIPY ligand, underscoring the critical role of Pt(II) in PDT amplification [106]. Mitochondria‐localized **20** induced membrane depolarization, disrupted microtubule assembly, selectively triggered apoptosis, and suppressed metastasis in lung carcinoma cells in vitro.

**FIGURE 7:**
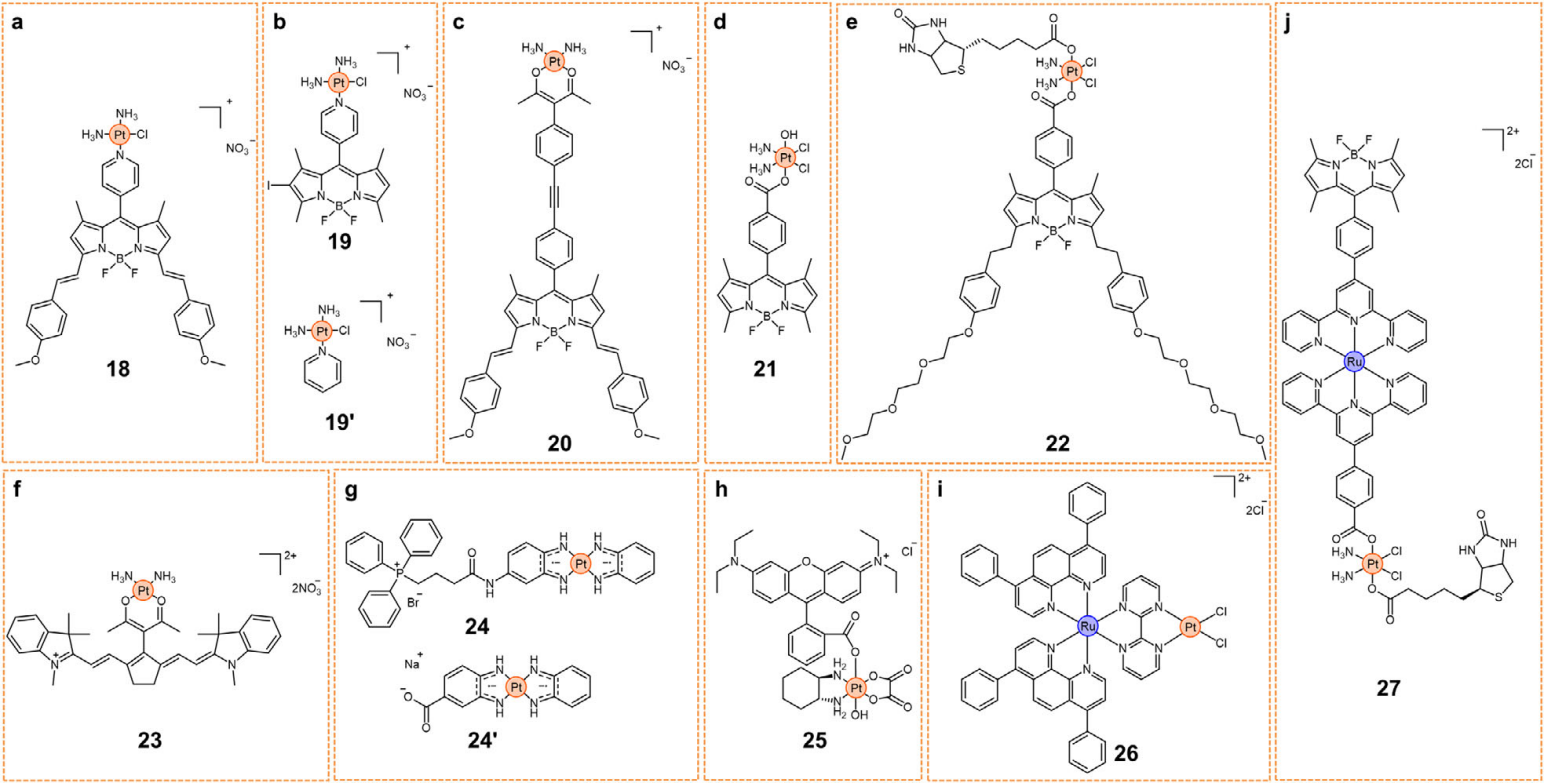
Mitochondria‐targeting photoactivatable Pt anticancer complexes and their non‐targeting analogues.

The photoactivated BODIPY ligand can also be incorporated into Pt(IV) prodrugs to enhance their activities. For example, complex **21** (Figure 7d) [107], a cisplatin‐based Pt(IV) prodrug featuring an axial BODIPY group, demonstrated potent light‐activated cytotoxicity in cancer cells, showing a remarkable SI of 41.8. Its efficacy stemmed from the mitochondria location, where it disrupted mitochondrial membrane integrity and induced cell death via a dual mechanism: released cisplatin and Pt‐enhanced PDT, which significantly outperformed the free BODIPY ligand in generating singlet oxygen. Similarly, a cisplatin‐based Pt(IV) prodrug (**22**, Figure 7e) featuring axial PEGylated distyryl‐BODIPY and biotin was designed to target cancer cells overexpressing biotin receptors [108]. Complex **22** accumulated significantly more in biotin receptor‐overexpressing A549 lung cancer cells than in noncancerous Beas‐2B cells, resulting in 41‐fold greater photocytotoxicity. Moreover, it also demonstrated potent PDT efficacy against multidrug‐resistant MDA‐MB‐231 cancer cells, indicating its potential to overcome resistance. The heavy‐atom effect from Pt enhanced the prodrug's singlet oxygen quantum yield beyond that of the free BODIPY ligand. Crucially, its preferential localization within mitochondria meant that light activation generated ROS directly at that site, resulting in mitochondrial membrane depolarization, microtubule disruption, and the efficient induction of apoptosis.

Heptamethine cyanine dyes are renowned for their strong NIR absorption, synthetic versatility, and organelle‐targeting properties, particularly toward mitochondria and lysosomes, making them valuable tools for bioimaging and PDT [109]. Mitra et al. developed a heptamethine cyanine‐Pt(II) conjugate (**23**, Figure 7f) as an NIR‐activatable theranostic agent [110]. Upon NIR irradiation, the Pt─O bond was cleaved to release active Pt(II) species—evidenced by elevated nuclear Pt content—and generate high levels of singlet oxygen. These concurrent effects acted synergistically, producing a 30–60‐fold increase in photocytotoxicity over dark conditions across cancer cells via DNA platination and ROS‐induced apoptosis. Pronounced mitochondrial (Pearson correlation coefficient (PCC): 0.94) colocalization and moderate lysosomal (PCC: 0.47) colocalization by the cyanine backbone further enhanced photocytotoxicity.

Triphenylphosphonium (TPP), a lipophilic cation with a delocalized positive charge, is widely utilized as a mitochondria‐targeting moiety due to its ability to traverse lipid bilayers and accumulate in mitochondria driven by the negative membrane potential [111, 112]. For instance, conjugation of TPP to a Pt diimine complex (**24**, Figure 7g) enhanced mitochondrial localization, significantly boosting singlet oxygen generation and photocytotoxicity compared to its non‐targeted analogue **24′** [113]. Notably, complex **24** maintained minimal cytotoxicity toward normal human liver cells (HL‐7702) under both dark and irradiated conditions, demonstrating cancer cell selectivity.

Conventional bioorthogonal catalysis for spatially controlled prodrug activation often suffers from inefficient substrate‐catalyst proximity [114]. To address this bottleneck, our group developed complex **25** (Figure 7h), an oxaliplatin‐derived Pt(IV) prodrug containing a Rhodamine B (RhB) photoswitch at the axial position [115]. This intramolecular design enforced proximity between the photoswitch and Pt(IV) core, boosting photoconversion efficiency 4.8 × 10^4^‐fold over external catalyst‐substrate systems. Consequently, complex **25** exhibited potent photocytotoxicity (IC_50_ 12‐fold lower than a mixture of dihydroxido‐Pt(IV) and PhB) and effectively overcame cisplatin resistance (RF = 0.8). Mitochondrial accumulation caused mtDNA damage and triggered intrinsic apoptosis independent of nDNA damage, further contributing to cell death.

Ruthenium (Ru) complexes, owing to their inherent lipophilicity and positive charge, may preferentially target mitochondria, similar to classic lipophilic cations like TPP and rhodamine 123 [116]. Exemplifying this, Mao, Tan, and coworkers engineered a heterobimetallic complex (**26**, Figure 7i) that integrated a mitochondria‐localizing Ru‐based PS with a Pt center [117]. The complex accumulated in mitochondria and, upon light irradiation, covalently anchored to and damaged mtDNA. This direct action caused mitochondrial dysfunction and triggered apoptosis, resulting in exceptional phototoxicity. This light‐dependent cytotoxicity effectively circumvented Pt resistance (RF = 2.0), as shown by its low IC_50_ values in both sensitive (0.9 ± 0.06 µM) and resistant (1.8 ± 0.06 µM) A549 lung cancer cells—a significant improvement over cisplatin (RF = 5.1). The mechanism—inducing mtDNA‐dependent efficacy and the mitochondrial‐driven apoptosis—translated to robust in vivo antitumor efficacy with low systemic toxicity. Similarly, Bera et al. designed a heterobimetallic Pt(IV)–Ru(II) prodrug (complex **27**, Figure 7j) featuring axial biotin and a BODIPY–Ru(II) PS [118]. Upon irradiation, it predominantly generated singlet oxygen (Type II) and a minor superoxide anion (Type I). This ROS generation, coupled with preferential uptake in cancerous A549 cells over noncancerous Beas‐2B cells, yielded highly selective photocytotoxicity, achieving a remarkable SI of 58.6. Mitochondrial localization further amplified photocytotoxicity by inducing photoactivated membrane depolarization, enhancing apoptosis, and suppressing A549 cell migration.

### 3.3 Lysosome‐Targeting Photoactivatable Pt Complexes

Lysosomes are single‐membrane organelles housing over 60 hydrolytic enzymes—including sulfatases, proteases, phospholipases, and phosphatases—and maintaining an acidic lumen (pH 4.5–5.0) critical for degrading macromolecules via endocytic, autophagic, and phagocytic pathways [119, 120, 121, 122]. Beyond waste processing, lysosomes are key regulators of cell signaling, migration, and programmed cell death [42]. Membrane integrity is pivotal: rupture releases enzymes, including cathepsins (and downstream caspase activation), into the cytosol, triggering apoptosis through controlled macromolecular degradation [123, 124, 125]. This inherent vulnerability, together with the observation that cancer cells can harbor up to three times more lysosomes than normal cells [126, 127], makes lysosomes compelling targets for photoactivatable Pt complexes. By preferentially accumulating in lysosomes, these complexes can induce membrane permeabilization and initiate organelle‐specific death pathways, thereby enhancing cytotoxicity in malignant cells while limiting damage to healthy tissues. Two primary targeting strategies are utilized to design lysosome‐targeting photoactivatable Pt complexes: (1) installing lysosome‐targeting moieties, typically basic amines such as morpholine, that become protonated in the acidic lysosomal lumen, promoting ion trapping due to reduced membrane permeability [128], and (2) leveraging endocytic uptake, whereby internalized complexes are trafficked to lysosomes as a terminal destination [129].

Xue et al. developed a photoactivatable monofunctional Pt(II) complex (**28**, Figure 8a) that preferentially localized in lysosomes via endocytosis [130]. Complex **28** exhibited minimal dark cytotoxicity but strong light‐activated cytotoxicity (> 13‐fold increase), generating ROS that depleted glutathione, destabilized lysosomal membranes, and facilitated nuclear translocation for DNA damage. Crucially, photoactivated lysosomal escape enabled complex **28** to potentially evade resistance mechanisms such as lysosomal sequestration, where carrier proteins mediate lysosomal entrapment and exocytosis of Pt drugs, limiting their nDNA accessibility [131].

**FIGURE 8:**
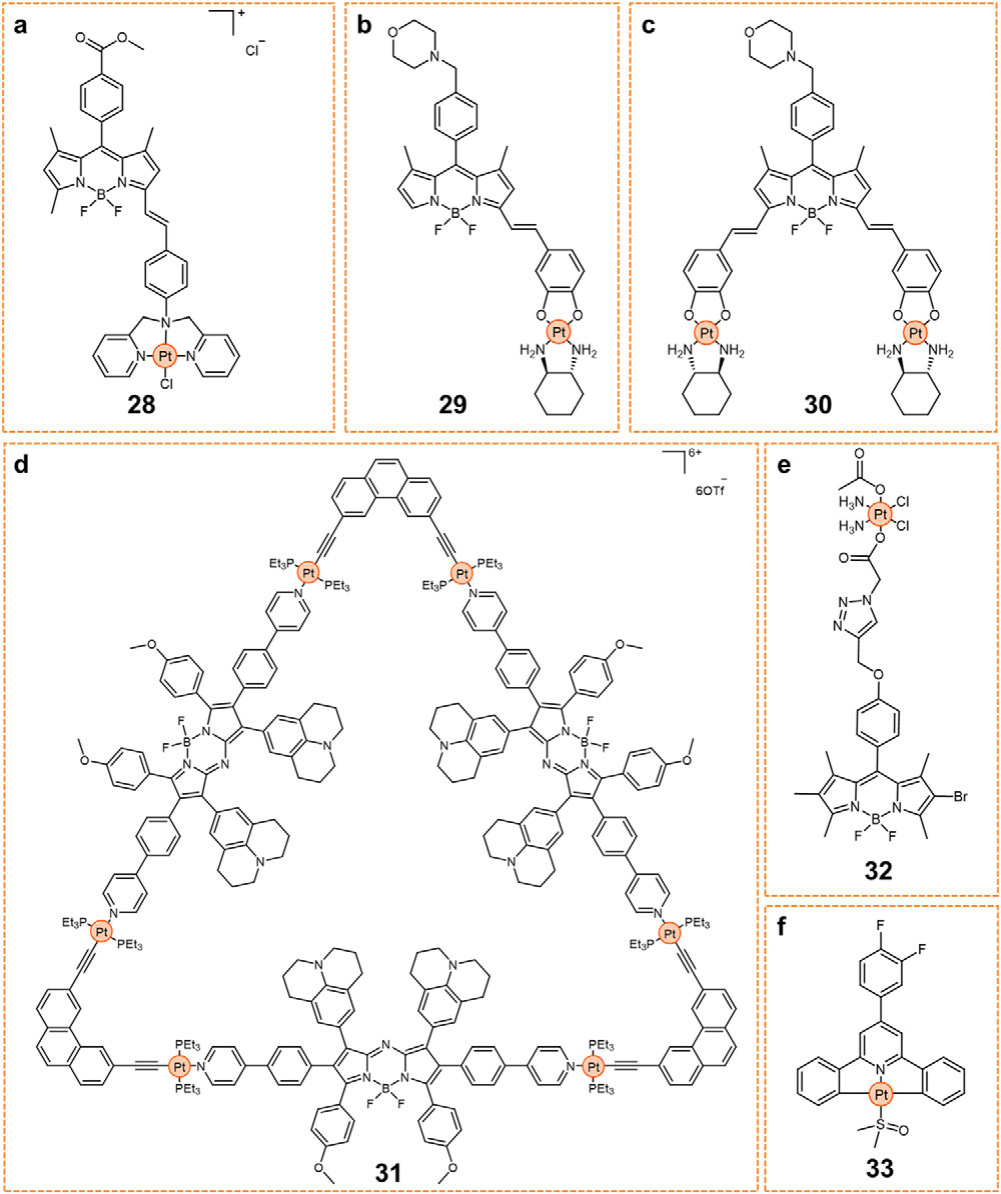
Lysosome‐targeting photoactivatable Pt anticancer complexes.

The morpholine group, a well‐established lysosome‐targeting moiety [132], conferred potent and selective anticancer properties to a near‐infrared Pt(II) complex (**29**, Figure 8b) [133]. The uptake of complex **29** via endocytosis facilitated lysosomal targeting that enabled singlet oxygen generation directly within the organelle, inducing apoptosis and yielding potent photoinduced cytotoxicity with negligible dark toxicity. Crucially, this strategy provided high cancer cell selectivity, evidenced by an approximately 10‐fold lower IC_50_ in malignant A549 versus nonmalignant HPL1D cells, a difference attributed to the lower lysosomal content in normal cells [131]. Expanding beyond mononuclear PDT agents, a binuclear Pt(II) complex (**30**, Figure 8c) was engineered for enhanced anticancer efficacy through targeted lysosomal disruption [134]. Its morpholine‐conjugated BODIPY ligand directed the complex to lysosomes (PCC: 0.91) via endocytosis, capitalizing on their higher abundance in cancer cells to achieve exceptional selectivity and a highly favorable therapeutic index [135]. This outcome of targeting resulted from NIR‐light‐induced lysosomal disruption: the photogenerated singlet oxygen permeabilized the lysosomal membranes, culminating in the catastrophic release of hydrolytic enzymes and the initiation of apoptosis exclusively in irradiated cancer cells [136].

Pt(II) metallacycles have shown promise in bioimaging and chemophototherapy due to favorable cellular uptake and prolonged tumor retention [137]. Sun, Sun, Stang, and colleagues developed a NIR‐activatable Pt(II) metallacycle (**31**, Figure 8d) as a potent ICD inducer for synergistic chemo‐photoimmunotherapy [138]. Complex **31** demonstrated robust, deep‐tissue ROS generation with superior antidiffusion and antiquenching properties under NIR excitation. It selectively accumulated in lysosomes, correlating with potent phototoxicity and cancer cell selectivity, as evidenced by its superior cytotoxicity versus cisplatin or oxaliplatin across cancer cell lines and a higher SI in the dark. Moreover, complex **31** overcame drug resistance, exhibiting greater potency in cisplatin‐resistant A549cisR cells than in cisplatin‐sensitive A549 cells, with an RF of 0.93. At low doses, it efficiently triggered damage‐associated molecular pattern (DAMP) release, achieving markedly greater ICD induction than oxaliplatin. In vivo, **31** potently induced ICD, stimulated anti‐tumor T cell responses, and depleted regulatory T cells, all with minimal systemic toxicity.

Spector et al. developed a dual‐action Pt(IV) prodrug (**32**, Figure 8e) integrating a PDT‐active BODIPY axial ligand for lysosome‐specific targeting [139]. The key advantage was its light‐activated potency: irradiation within the lysosome triggered organelle disruption and concurrently reduced the prodrug, releasing cisplatin. This process culminated in a more than 100‐fold increase in antiproliferative activity compared to its inactive state in the dark.

To address challenges in four‐photon absorption (4PA) material design, Tian's group synthesized a 4PA‐active Pt(II) complex (**33**, Figure 8f) by incorporating a functionalized C^N^C ligand [140]. This complex exhibited NIR‐II phosphorescence and a notably large 4PA cross‐section, demonstrating strong lysosomal colocalization, likely attributed to its interaction with lecithin, a key lysosomal membrane phospholipid [141]. Complex **33** displayed potent and selective cytotoxicity, significantly outperforming cisplatin. In vivo, it effectively inhibited tumor growth without body weight loss or cisplatin‐associated toxicity, underscoring its superior therapeutic index and tumor specificity.

### 3.4 ER‐Targeting Photoactivatable Pt Complexes

The ER, the largest membranous organelle in eukaryotic cells, comprises structurally and functionally distinct domains: the ribosome‐studded rough ER (RER), which is responsible for protein synthesis, folding, and trafficking, and the smooth ER (SER), which regulates lipid synthesis, calcium homeostasis, and redox signaling [142, 143, 144]. Beyond its biosynthetic functions, the ER serves as a central hub for stress sensing, primarily through the unfolded protein response (UPR), which is activated by disturbances such as Ca^2+^ dysregulation, oxidative stress, or protein misfolding [145, 146]. Persistent ER stress can initiate pro‐apoptotic pathways, including mitochondrial cytochrome c release, caspase activation, and upregulation of stress markers, ultimately culminating in programmed cell death [147, 148]. PDT and PACT exploit these mechanisms by generating ER‐localized ROS, disrupting Ca^2+^ homeostasis, and amplifying ER stress to induce tumor‐selective apoptosis. Consequently, targeting the ER presents a promising strategy to enhance anticancer efficacy.

Leveraging curcumin's inherent ER‐targeting capability [149, 150], a BODIPY‐tagged Pt(II) curcumin complex (**34**, Figure 9a) was developed for ER‐targeted PDT [151]. Upon red light irradiation, complex **34** generated significant ROS and demonstrated excellent light‐dependent cytotoxicity, exhibiting a 12.5‐fold increase in A549 cells compared to dark conditions, while maintaining comparable photocytotoxicity even in the multidrug‐resistant MDA‐MB‐231 cell line. It localized preferentially in the ER, inducing oxidative stress and disrupting ER functions. Partial mitochondrial localization further contributed to apoptotic death through ER stress and mitochondrial membrane depolarization.

**FIGURE 9:**
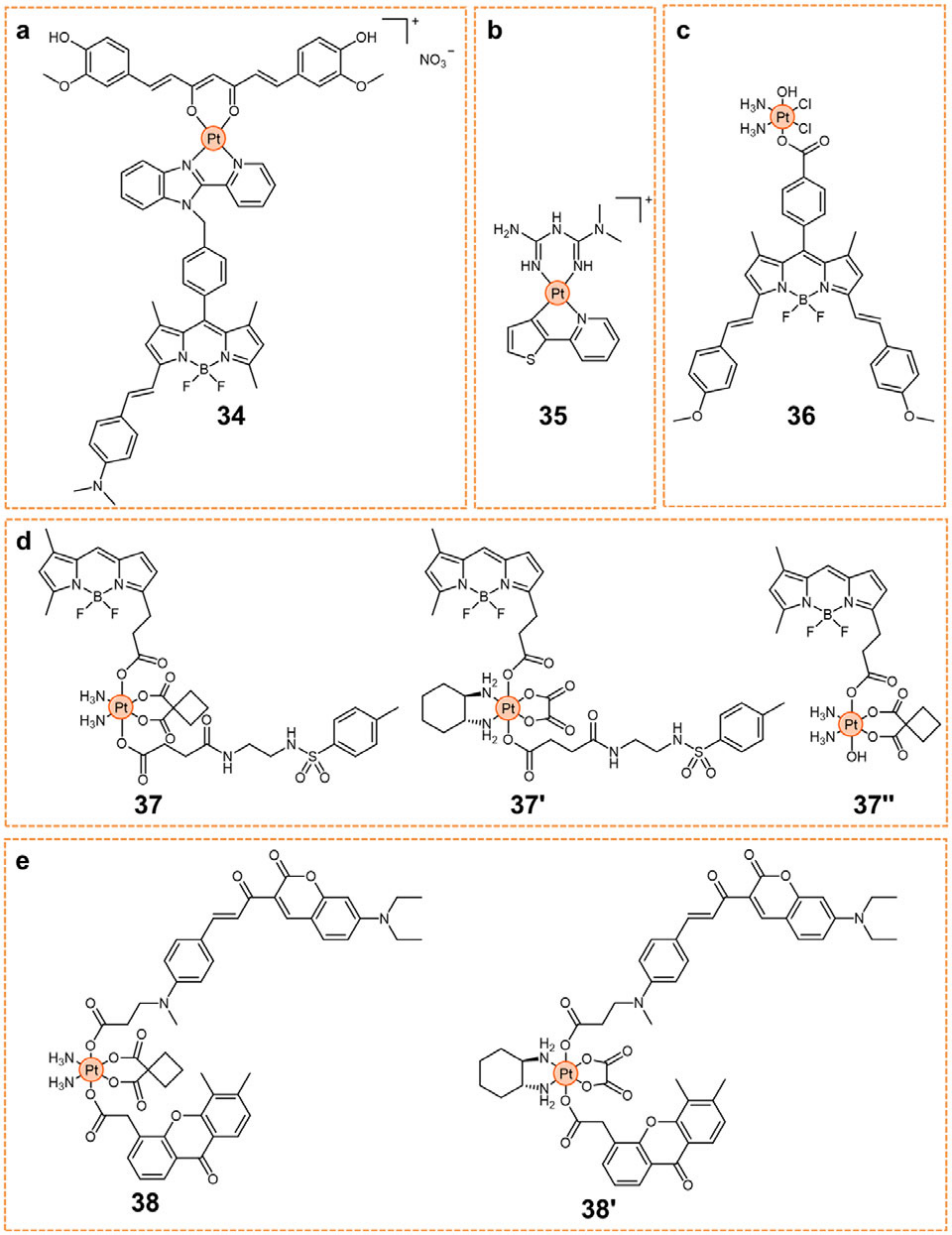
ER‐targeting photoactivatable Pt anticancer complexes and their nontargeting analogues.

Metformin (Met), an FDA‐approved antidiabetic drug with recognized anticancer properties [152], was incorporated into an NIR‐phosphorescent Pt(II) complex (**35**, Figure 9b) by Yang et al. [153]. Complex **35** preferentially accumulated in the ER and underwent energy‐dependent internalization in HeLa cells. Light irradiation enhanced its activity, yielding a phototoxic index (PI, defined as IC_50_ in the dark/IC_50_ under irradiation) of 3.3 in A549 lung cancer cells. The complex exhibited an SI of 1.9 in human normal lung fibroblast‐like cells (HLF), outperforming cisplatin's SI of 1.1. Mechanistically, it promoted covalent interactions with bovine serum albumin, induced ROS generation, caused mitochondrial membrane potential loss, triggered cell cycle arrest, and exhibited anti‐metastatic activity, collectively leading to apoptosis.

Although limited research exists on ER‐targeted Pt(IV) complexes for PDT, a red light‐activatable Pt(IV)–BODIPY prodrug (complex **36**, Figure 9c) was designed for combined ER‐targeted chemo‐PDT [154]. This prodrug exhibited high dark stability but underwent rapid photoactivation in redox‐mimicking conditions. Upon activation, complex **36** displayed potent photocytotoxicity in cancer cells with minimal cytotoxicity in noncancerous cells, outperforming cisplatin by approximately 100‐fold. Its preferential ER accumulation (PCC: 0.89) and significant mitochondrial colocalization (PCC: 0.79) effectively avoided nuclear localization, bypassing the NER mechanism that compromises cisplatin's activity. Mechanistically, complex **36** caused cell cycle arrest, induced ER stress, and triggered mitochondrial dysfunction, collectively leading to apoptosis.

To address limitations like premature reduction and low photocytotoxicity in oxaliplatin‐based Pt(IV) prodrugs, Yao, Yuan, and coworkers engineered a carboplatin‐based Pt(IV) prodrug (complex **37**, Figure 9d) functionalized with an ER‐targeting phenyl sulfonamide and a BODIPY PS [155]. Complex **37** demonstrated superior dark stability over its oxaliplatin‐based analogue (**37′**) and efficiently generated singlet oxygen upon light exposure, undergoing rapid, reductant‐accelerated activation. It showed significantly enhanced photocytotoxicity compared to **37′** and the nontargeting analogue **37″**, attributed to rapid photoactivation and specific ER targeting (PCC = 0.96). Complex **37** effectively overcame drug resistance and exhibited 4‐fold and 31‐fold greater potency than carboplatin in A549 and A549cisR cells, respectively.

While red and NIR light (600–1400 nm) enable deeper targeting, many PACT agents lack NIR responsiveness, and oxygen‐dependent PDT drugs fail under hypoxic conditions [156]. To address these challenges, our group developed NIR‐activatable Pt(IV)‐coumarin photooxidants **38** and **38′** (Figure 9e) [157]. These photooxidants preferentially localized to the ER, where, under irradiation, they underwent ER‐specific, oxygen‐independent, two‐photon photoreduction. This process generated potent oxidative bursts within the ER, causing robust oxidation of biomolecules, generating ROS, lipid peroxides, and protons, which led to oxidative bursts, ER stress, disrupted intracellular pH homeostasis, and ICD via nonclassical necrosis. This ER‐focused mechanism conferred four exceptional outcomes: (1) unmatched photopotency (>461‐fold over standard chemotherapeutics) without dark cytotoxicity, (2) complete abrogation of Pt resistance (RFs 0.95–1.06), (3) maintenance of full potency in hypoxia, confirming oxygen independence, and (4) stimulation of a potent antitumor immune response that suppressed both tumor growth and metastasis in murine models.

## 4 Summary and Outlook

### 4.1 Summary

PDT and PACT have emerged as promising anticancer modalities owing to their noninvasiveness, spatiotemporal precision via light activation, and robust tumor‐suppressive effects. Light serves as an external trigger that enables exquisite control over treatment timing and location, restricting cytotoxicity to irradiated tumors while sparing adjacent healthy tissues. These light‐based approaches offer a compelling alternative to conventional Pt‐based chemotherapy, which is often limited by systemic toxicity and drug resistance. Recent advancements integrating Pt agents into PDT and PACT systems—particularly via cancer cell membrane protein‐ and organelle‐targeting strategies—harness light‐triggered mechanisms to amplify localized therapeutic outcomes, thereby providing innovative solutions to these challenges. By exploiting the unique physiological roles of cancer cell membrane proteins and subcellular structures (e.g., nucleus, mitochondria, lysosomes, and ER), these strategies enhance therapeutic precision by directing photochemical reactions to cancer cells or specific intracellular sites. This review outlines current approaches to developing targeted phototherapeutic Pt complexes, emphasizing their structural design, mechanisms of action, and improved therapeutic effects compared to nontargeted analogues. Targeting cancer cells and critical organelles enhances the activation of lethal cell death pathways and enables the selective destruction of malignant structures, offering significant advantages over conventional nonspecific methods. Such cancer cell membrane protein‐targeting and organelle‐specific targeting strategies not only reduce required drug doses but also mitigate the risks of MDR, tumor recurrence, and adverse effects. Furthermore, these advanced complexes exhibit enhanced anticancer activity, the capacity to circumvent drug resistance, and improved cancer cell selectivity relative to traditional Pt agents. Thus, they represent a promising strategy for overcoming the limitations of existing Pt drugs and advancing the development of targeted phototherapeutic Pt complexes for precise cancer therapy.

Upon irradiation, targeted photoactivable Pt complexes induce cancer cell death or organelle‐specific cell death through two principal mechanisms: direct PDT, which generates localized ROS, particularly singlet oxygen, and photoreduction of Pt(IV) to cytotoxic Pt(II), often accompanied by the release of ligand fragments (Table 1). Both mechanisms can be harnessed to produce spatially confined phototoxicity at the intended subcellular site. For example, nuclear‐directed ROS directly damages DNA, activating ICD or senescence; mitochondria‐localized ROS oxidizes mtDNA, depolarizes membrane potential, and triggers intrinsic apoptosis via cytochrome c release. This potent ROS generation is further strengthened by Pt coordination, which serves as a general photophysical enhancer through the heavy‐atom effect. Specifically, Pt can accelerate intersystem crossing, increase triplet‐state population, elevate singlet oxygen quantum yield, and red‐shift absorption into the therapeutic red/NIR window. In Pt(IV) PACT agents, axial‐ligand design further enables controlled photoreduction under irradiation, releasing bioactive Pt(II) and/or ROS. Collectively, these rationally integrated strategies, which combine organelle‐directed targeting ligands with Pt‐mediated photophysical tuning, help overcome the core limitations of conventional PDT (off‐target ROS, shallow tissue penetration) and platinum chemotherapy (monomodal cell death pathways and limited therapeutic efficacy).

**TABLE 1:** Cancer cell membrane protein‐/organelle‐targeted Pt complexes in photodynamic/photoactivated therapy: Mechanisms, ROS localization effects, and influences of Pt coordination.

Targeted complex	Photomechanism and activation summary	Organelle localization alters ROS diffusion and damage pathways	Pt coordination influences photophysical processes	Ref
*Cancer cell membrane protein* (receptor‐mediated uptake; complexes **2–11**)	Receptor‐mediated endocytosis for selective cancer cell uptake (FR‐/integrin‐/GLUT‐overexpressed); visible/red‐light activation of Pt(IV) photoreduction to Pt(II) + ligand fragments or direct Type II PDT (^1^O_2_ from PS ligands); multiple cytotoxic species.	Localization confines ROS to endocytic vesicles/membrane, thereby enhancing anticancer efficiency. Enhanced selective accumulation in receptor‐overexpressing cancer cells.	Pt heavy‐atom effect (spin‐orbit coupling) enhances ISC in BODIPY/porphyrin/curcumin ligands (**4**, **5**, **9–11**), elevating ^1^O_2_ quantum yield; axial coordination in Pt(IV) (**2**, **6**, **8**) enables controlled photoreduction (visible light → Pt(II) + ROS byproducts). Conjugation improves photostability and red‐shifted absorption for deeper penetration versus free PS.	[52, 53, 55, 64, 65, 66, 70, 71, 72]
*Nucleus* (complexes **12–17**)	NLS/TPA/porphyrin‐driven nuclear targeting; blue/visible light: Pt(IV) photoreduction to Pt(II) (DNA binding) or direct PDT (ROS); DNA damage/ICD/senescence.	Nuclear confinement directs short‐lived ROS straight to DNA (intercalation/cleavage), bypassing cytoplasmic diffusion losses.	Pt(II) porphyrin/triphenylamine (**13–16**): heavy‐atom effect + intercalation rigidify the complex, boost ISC/^1^O_2_ and DNA affinity. Pt(IV) axial photocages (**12**) enable controlled reduction to active Pt(II).	[86, 87, 88, 90, 91, 92]
*Mitochondria* (complexes **18–27**)	TPP/RhB/heptamethinecyanines‐driven Mt‐targeting; red/NIR light: PS → ^1^O_2_ (or •OH), Pt(IV) photoreduction; mtDNA damage, Mt membrane potential (ΔΨm) collapse.	ΔΨm drives accumulation; localized ROS (diffusion‐limited) oxidizes mtDNA/cardiolipin → rapid Δ*Ψ*m collapse, cytochrome c release, and intrinsic apoptosis/necrosis—independent of nuclear DNA.	Pt heavy‐atom effect in BODIPY/cyanine/Ru‐based conjugates (**18–20**, **23**, **26**) dramatically increases ISC and ROS quantum yield under red/NIR light; axial BODIPY/RhB/Ru‐based derivative in Pt(IV) (**21**, **22**, **25**, **27**) enables mitochondria‐specific photoactivation.	[104, 105, 106, 107, 108, 110, 113, 115, 117, 118]
*Lysosome* (complexes **28–33**)	Red/NIR/green‐light PDT; lysosomal trapping → photoinduced lysosomal membrane permeabilization (LMP); ROS → escape to nucleus/DNA damage or ICD in some.	Lysosomal trapping in dark: photo‐ROS causes LMP → cathepsin release, caspase activation/apoptosis, or nuclear translocation; exploits cancer lysosome abundance for selectivity.	Pt coordination to BODIPY/catecholates (**29–30**, **33**) enhances ISC for NIR/red activation; cyclometalated Pt(II) (**28**, **31**) improves lysosomal affinity while boosting triplet/^1^O_2_ quantum yield; photolabile Pt(IV) (**32**) allows controlled release inside acidic lysosomes.	[130, 133, 134, 138, 139, 140]
*ER* (Complexes **34–38**)	Curcumin/BODIPY/metformin for ER‐targeting; red/NIR light → ^1^O_2_ + •OH (type I/II); O_2_‐independent in Pt(IV); ER stress induction.	ER localization confines ROS to a protein‐rich environment → activates UPR and Ca^2+^ efflux → mitochondrial secondary damage → amplified mitochondrial apoptosis/ICD.	Pt‐curcumin/BODIPY (**34**, **36**): heavy‐atom effect boosts ISC for dual ROS (^1^O_2_ from BODIPY, •OH from curcumin); Pt(IV) axial ligands (**36–38**) enable photoreduction directly at ER, boosting quantum efficiency and photostability versus metal‐free PS.	[151, 153, 154, 155, 156]

### 4.2 Outlook I: The Design Principles for Selecting Cancer Cell Membrane Protein or a Specific Organelle to Target

The cancer cell and subcellular localization of photoactivatable Pt complexes can be rationally tailored to achieve specific therapeutic goals, such as enhanced cancer cell selectivity, improved cytotoxicity, and reduced resistance. The examples surveyed here therefore furnish valuable strategic guidance for the future design of precision cancer therapeutics (Table 2). For example, when the primary objective is to enhance cancer cell selectivity, direct cancer‐cell targeting via receptor‐mediated uptake or lysosomal accumulation may be effective, particularly because malignant cells typically contain more lysosomes than normal cells. When the goal is to increase cytotoxicity, targeting the ER, the largest organelle in the cell, may be advantageous because it can induce widespread proteotoxic stress. If both enhanced cytotoxicity and resistance circumvention are desired, nuclear targeting is especially attractive, as direct DNA damage constitutes the canonical mechanism of Pt agents and can both increase therapeutic potency and bypass common resistance pathways. Mitochondrial targeting likewise represents an attractive dual‐purpose strategy because it may simultaneously improve selectivity and potency by disrupting membrane potential and damaging mtDNA. Furthermore, mitochondrial localization offers particular advantages for Pt‐based phototherapy. The organelle's marked sensitivity to ROS, together with its central role in intrinsic apoptotic signaling, enables potent photocytotoxicity even at modest light doses. These cancer cell membrane protein‐ and organelle‐specific approaches delineate a coherent direction for the development of the next generation of photoactivatable Pt therapeutics; nevertheless, they remain directional suggestions that merit rigorous experimental validation through continued multidisciplinary investigation.

**TABLE 2:** Key pharmacological parameters and therapeutic outcomes of cancer cell membrane protein‐/organelle‐targeted photoactivatable Pt complexes.

Targeting	Representative complex	ROS quantum yield^a–d^	Light dose (J/cm^2e^/mW/cm^2f^)	Irradiation wavelength (nm)	Irradiation time (min)	Cell line	Complex	IC_50_ value (µM)	Therapeutic performance	Key outcome	Ref
*Cancer cell membrane protein (receptor‐mediated uptake)‐targeting*	**9**	0.58^b^	30^e^	600−720	40	A549^g^	**9**	2.3 ± 0.1	>43.48 (SI^j^)	*Enhanced cancer‐cell selectivity*	[70]
						HPL1D	**9**	>100			
	**10**	n.d.^a^	10^e^	400−700	60	A549^g^	**10**	20.8 ± 0.1	3.50 (SI)		[71]
						HPL1D	**10**	72.9 ± 0.8			
*Nucleus‐targeting*	**12**	n.d	8^f^	450	60	HCT116^h^	**12**	1.3 ± 0.3	68.69 (FI^k^)	*Improved cytotoxicity and reduced resistance*	[86]
						HCT116	**12'**	89.3 ± 5.6			
						A549^]^	**12**	6.9 ± 1.1	0.58 (RF^l^)		
						A549CisR	**12**	4.0 ± 0.5			
	**14**	0.81^c^	40^f^	425	15	HepG2^h^	**14**	1.2 ± 0.1	20.92 (FI)		[88]
						HepG2	**14'**	25.1 ± 1.2			
						A549^h^	**14**	0.79 ± 0.08	0.42 (RF)		
						A549CisR	**14**	0.33 ± 0.07			
*Mitochondria‐targeting*	**21**	0.29^b^	2.4^f^	400–700	60	A549^g^	**21**	1.1 ± 0.3	41.82 (SI)	*Enhanced cancer‐cell selectivity and improved cytotoxicity*	[107]
						HPL1D	**21**	46 ± 1			
						A549 (dark)	**21**	>50	>45.45 (PI^m^)		
						A549 (light)	**21**	1.1 ± 0.3			
	**27**	0.64^b^	5^e^	400−700 nm	15	A549^[g]^	**27**	0.022 ± 0.004	58.64 (SI)		[118]
						Beas‐2B	**27**	1.29 ± 0.20			
						A549 (dark)	**27**	> 100	> 4545 (PI)		
						A549 (light)	**27**	0.022 ± 0.004			
*Lysosome‐targeting*	**29**	n.d.	30^e^	600–720	60	A549^g^	**29**	4.1 ± 0.3	10.98 (SI)	*Enhanced cancer‐cell selectivity*	[133]
						HPL1D	**29**	45.0 ± 0.1			
	**30**	n.d.	30^e^	600−720	15	A549^g^	**30**	0.7 ± 0.2	88.71 (SI)		[134]
						HPL1D	**30**	62.1 ± 1.4			
*ER‐targeting*	**36**	0.52^d^	30^e^	600–720	20	HeLa (dark)^g^	**36**	>100	>172 (PI)	*Improved cytotoxicity*	[154]
						HeLa (light)	**36**	0.58 ± 0.02			
	**37**	0.057^c^	2.3^f^	green light	30	HepG2^i^	**37**	9.5 ± 0.4	>16.84 (FI)		[155]
						HepG2	**37''**	>160			

^[a]^Not determined.

The ROS quantum yield was determined relative to the standard:

^[b]^Rose bengal.

^[c]^[Ru(bpy)_3_]Cl_2_].

^[d]^Methylene blue.

The unit of light dose:

^[e]^J/cm^2^.

^[f]^mW/cm^2^.

Cytotoxicity was evaluated by treating cells with the corresponding complex, followed by irradiation and post‐incubation, with total incubation times for:

^[g]^24 h.

^[h]^48 h.

^[i]^72 h.

^[j]^Selectivity index, SI = IC_50_ in normal cells/IC_50_ in cancer cells.

^[k]^Fold increase, FI = IC_50_ of nontargeted analogue/IC_50_ of targeted complex.

^[l]^RF = IC_50_ in resistant cells/IC_50_ in sensitive cells.

^[m]^PI = IC_50_ in the dark/IC_50_ under irradiation.

The strategic selection of cancer cell receptors and subcellular organelle targets for photoactivated metal complexes in PDT or PACT can also be guided by the distinct pathophysiological and molecular vulnerabilities of specific cancer types. This cancer‐type‐centered design principle exploits hallmark tumor traits to maximize selective phototoxicity, overcome resistance, and enhance clinical translation potential (Table 3) [190, 191]. In cancers that overexpress cell‐surface receptors, the receptor‐targeting takes advantage of ligand‐mediated endocytosis to promote preferential uptake while largely sparing normal tissues with lower receptor expression [161]. This strategy is particularly relevant to head and neck, cervical, bladder, and oesophageal cancers, in which elevated EGFR expression correlates strongly with aggressive behavior and poor clinical outcomes; accordingly, directing complexes to membrane‐associated EGFR provides a rational means of selectively eradicating these cancer cells [162]. Cancers characterized by rapid proliferation or defective DNA repair are well suited to nuclear targeting, which inflicts direct photodamage to DNA [164, 166]. Small cell lung cancer (SCLC) provides an instructive example because it typically exhibits a high mutational burden, frequent loss of TP53 and RB1, and amplification of MYC family members, all of which contribute to substantial genomic instability. As a result, SCLC is particularly susceptible to interventions that engage the DNA damage response (DDR) pathway and selectively eradicate genomically unstable cells [165]. Metabolically reprogrammed solid tumors, characterized by the Warburg effect and elevated Δ*Ψ*m, exhibit pronounced mitochondrial dependence and are therefore highly amenable to mitochondrial‐targeted therapies. Such strategies induce membrane permeabilization, bioenergetic collapse, redox imbalance, and apoptosis or ferroptosis [170, 186, 192]. Colorectal cancer (CRC) relies precisely on these mitochondrial adaptations to survive hypoxia and therapeutic stress. Mitochondrial targeting can therefore dismantle these protective mechanisms and drive selective cancer cell death [177]. Cancers marked by aggressive autophagy dependence or MDR are particularly vulnerable to lysosomal targeting, which promotes membrane permeabilization and cathepsin release [178, 179]. Because MDR often develops through enhanced efflux pump activity and lysosomal sequestration of chemotherapeutic agents, lysosome‐targeted membrane permeabilization can resensitize refractory cancer cells, including those in adriamycin‐resistant MCF‐7 breast and MDR lung cancer cells, to conventional chemotherapy [127, 184]. Cancers with particularly high protein synthesis demands or immune‐evasive properties are also responsive to ER targeting, which induces severe stress, activates the UPR, promotes Ca^2+^ efflux, and triggers ICD or pyroptosis [186]. Breast cancer provides a representative example, particularly in estrogen receptor α‐positive (ERα+) and triple‐negative subtypes, in which cells often exploit ER stress and UPR signaling to promote progression, metastasis, and therapy resistance in a subtype‐specific manner. Direct disruption of ER homeostasis can therefore overwhelm this adaptive capacity, induce unresolved proteotoxic stress, and restore treatment sensitivity [189]. These cancer‐type‐tailored strategies have been validated in two‐ and three‐dimensional culture systems, xenografts, and emerging clinical data [156, 193, 194, 195]. These cancer‐type‐tailored strategies may serve as general design guidelines for diverse targeted metal complexes and are particularly well suited to photoactivated platinum systems, whose inherent spatiotemporal activation control minimizes off‐target toxicity. Nonetheless, Table 3 is presented as a conceptual guideline and hypothesis‐generating framework: the principles therein are speculative, intended to inspire rational design rather than to constitute empirically validated rules, and merit rigorous experimental confirmation through continued multidisciplinary investigation.

**TABLE 3:** Cancer cell membrane protein‐ and organelle‐targeted photoactivated metal complexes—cancer‐type‐specific design principles.

Target	Possible cancer types	Cancer characteristics	Rationale for effective killing by organelle targeting	Ref
*Cancer cell membrane proteins (receptors on the membrane, higher uptake)*	Head and Neck/Cervical/Bladder/Oesophageal/Colorectal (EGFR^+^). Breast (MCF‐7) (FR^+^). Glioma/Breast (MDA‐MB‐231) (Integrin αvβ3^+^)	Overexpression of surface receptors (folate receptors, or vitamin transporters, EGFR overexpression, integrin‐driven invasion/angiogenesis). High angiogenic, invasive, and metastatic potential with receptor‐driven signaling.	Selective receptor binding enables ligand/antibody‐mediated endocytosis and markedly higher intracellular accumulation; localized photoactivation disrupts membrane integrity or signaling with minimal systemic exposure and maximal tumor selectivity.	[158, 159, 160, 161, 162]
*Nucleus*	Lung (small cell lung cancer SCLC); BRCA‐mutant breast; proliferative colorectal; cervical (HeLa); highly proliferative carcinomas	High proliferation rate, genomic instability, or defective DNA repair (BRCA mutations). Transcriptional/replicative dependence in rapidly dividing or repair‐deficient tumors. Cells are vulnerable to replication/transcription blockade.	Direct DNA photodamage (intercalation/groove binding or ROS) halts replication/transcription and induces cell‐cycle arrest/apoptosis; effective in repair‐deficient cells.	[163, 164, 165, 166, 167, 168, 169]
*Mitochondria*	Colorectal; lung (A549); breast (MCF‐7, MDA‐MB‐231, triple‐negative); liver (HepG2); cervical (HeLa); prostate; hepatocellular carcinoma (HCC)	Elevated Warburg‐effect metabolic reprogramming in these metabolically active/resistant tumors, elevated Δ*Ψ*m, high ROS sensitivity, apoptosis induction, and hypoxic cores common in solid tumors.	Localized ROS generation collapses mitochondrial membrane potential, releases cytochrome c, and activates caspase‐dependent intrinsic apoptosis. Effective even under hypoxia or in resistant phenotypes due to its central role in energy and death pathways.	[170, 171, 172, 173, 174, 175, 176, 177]
*Lysosome*	Many multi‐drug resistance (MDR; Drug‐resistant esophageal/Adriamycin‐resistant MCF‐7/multidrug‐resistant lung cancer cells). Aggressive/metastatic subsets (HCC). Colorectal (CT26)	Elevated autophagic flux and lysosomal biogenesis for survival under hypoxia/nutrient/therapy stress are common in aggressive, metastatic, or therapy‐resistant phenotypes.	Induces lysosomal membrane permeabilization, cathepsin release, caspase activation or necrosis, and blocks protective autophagy; bypasses certain resistance mechanisms and exerts anti‐metastatic effects.	[127, 178, 179, 180, 181, 182, 183, 184]
*ER*	Breast [estrogen receptor α‐positive (ERα+) and triple‐negative subtypes]; lung (A549, NSCLC); prostate; colorectal; head and neck squamous cell carcinoma	High protein biosynthetic/secretory demand or mutational burden; immunosuppressive “cold” microenvironments; ER‐stress sensitivity in secretory or rapidly proliferating tumors; UPR hypersensitivity.	Photoinduced ROS induces a severe UPR, Ca^2+^ efflux, protein oxidation/cross‐linking, pyroptosis, and ICD, activating antitumor immunity—highly effective in secretory or immunoevasive tumors.	[185, 186, 187, 188, 189]

Effective cancer cell‐ and organelle‐targeting relies on the strategic use of ligands that exploit specific receptors and subcellular biomarkers or the unique characteristics of particular organelles (Figure 10). For instance, folic acid, RGD peptides, and glucose derivatives target the cancer cell receptors by binding to overexpressed FRs, integrins, and GLUT1, respectively. NLS (e.g., R_8_K) and triphenylamine derivatives are employed for nuclear delivery, while TPP and rhodamine B derivatives leverage their lipophilicity and cationic charge to achieve mitochondrial accumulation. Morpholine groups direct Pt complexes to lysosomes with high specificity, whereas phenyl sulfonamide groups enable ER targeting. These targeting strategies play a pivotal role in designing organelle‐targeted Pt complexes for precise PDT and PACT. Despite substantial advancements, challenges persist as lysosome‐ and ER‐targeted complexes remain comparatively underdeveloped, likely due to a limited availability of tailored ligands. Advancing in these areas requires both expanding the repertoire of targeting ligands—especially for ER and lysosomes—and systematically elucidating subcellular trafficking processes to enhance therapeutic precision. Moreover, improving the selectivity of anticancer agents necessitates deeper insights into the pathophysiological differences between organelles in normal and malignant cells, which will refine targeting strategies and help mitigate chemoresistance [196]. Addressing these gaps is crucial to unlocking the full therapeutic potential of organelle‐directed Pt complexes.

**FIGURE 10:**
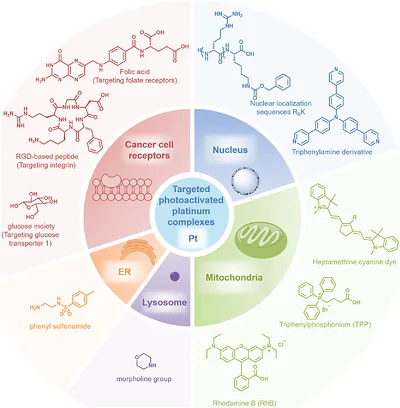
The structure of targeting groups that can direct Pt into cancer cells or different organelles.

### 4.3 Outlook II: The Challenges of Clinical Translation of Targeted Pt‐Based PDT and PACT Complexes

Despite their promise as innovative cancer therapies, the clinical translation of targeted Pt‐based PDT and PACT agents faces significant challenges that extend beyond simply incorporating receptor‐ and organelle‐targeting ligands. First, some targeted phototherapeutic Pt complexes exhibit relatively poor stability and premature activation—manifested through susceptibility to hydrolysis, reduction, ligand exchange reactions with water or thiol‐containing biomolecules, and organelle‐specific conditions such as mitochondrial reducing environments or lysosomal acidity—which can lead to unintended decomposition and off‐target effects. This instability not only exacerbates toxicity but also raises serious long‐term biosafety concerns, especially if the agents are not efficiently metabolized or excreted. The typically poor aqueous solubility of these complexes presents a major delivery challenge, often necessitating formulation with solubilizing excipients for systemic administration. These excipients, however, may introduce their own adverse effects, further complicating biosafety profiles [197]. More fundamentally, organelle targeting introduces layered complexity: Pt complexes must first accumulate in tumors and then localize precisely within organelles while preserving phototherapeutic efficacy. Tumor targeting is therefore a prerequisite; without it, non‐specific distribution to healthy tissues increases the risk that incidental light exposure (e.g., scattering, reflections, or irradiation of tumor margins) will activate the agents and cause collateral damage. Furthermore, many studies remain confined to cell‐based assays, making translational relevance uncertain; even when animal testing is conducted, preclinical testing often relies on short‐term murine models that are inadequate for evaluating chronic toxicity and long‐term biosafety. To overcome these barriers, future efforts should prioritize rational drug design, advanced delivery strategies, and comprehensive in vivo tests. Key directions include: (1) molecular optimization to enhance stability and reduce nonspecific reactivity, (2) incorporation into nanoformulations (e.g., liposomes and polymeric nanoparticles) to improve solubility and prolong circulation, and/or use of FDA‐approved solubilizing agents to enhance safety, (3) active targeting through conjugation to tumor‐homing ligands (e.g., peptides and antibodies) to improve specificity, and (4) extensive animal evaluations to validate therapeutic efficacy. Systematic preclinical animal studies, particularly comparative pharmacokinetic and toxicological profiling, are essential to identify suitable organelles for different purposes. Crucially, rigorous and long‐term in vivo biodistribution and toxicity studies will be essential to de‐risk these innovative agents and pave the way toward clinical trials.

The clinical translation of targeted phototherapeutic Pt complexes is also impeded by the general challenges common to PDT and PACT. One critical obstacle is optimizing the photoactivation wavelength of targeted Pt complexes to align with tumor depth: deep‐seated lesions necessitate red or NIR light for sufficient tissue penetration, yet water absorption increases substantially at longer wavelengths (> 900 nm, with peaks at 960, 1440, 1950 nm), restricting the usable range for PDT and PACT within the NIR‐II window. In contrast, superficial tumors, such as gliomas and non‐muscle‐invasive bladder cancer, require precisely controlled shallow activation to avoid damaging underlying healthy tissue [18]. For both organelle‐targeted Pt‐based PDT and PACT, achieving efficient, controlled, deep‐tissue activation presents a fundamental hurdle. Current strategies to overcome this include developing organelle‐targeted Pt complexes that can be activated by longer‐wavelength light, as well as designing systems responsive to other stimuli such as ultrasound or x‐rays [198]. Additionally, the efficacy of organelle‐targeted Pt‐based PDT is constrained by tumor hypoxia, which impedes oxygen‐dependent Type II PDT efficacy, driving the development of PSs that preferentially operate via oxygen‐independent Type I pathways or PACT mechanisms. A notable contribution of this review is its systematic survey of diverse PS platforms (Table 4), furnishing a versatile repertoire of structural motifs and mechanistic insights for the rational design of future photoactivatable Pt‐based therapeutics. Exemplified by BODIPY, porphyrin, and coumarin derivatives, these ligands exhibit distinct photophysical and photochemical properties that enable tailored ROS generation through Type I, Type II, or PACT mechanisms. These platforms also differ in their relevance under hypoxia: Type II processes are severely restricted, whereas Type I and PACT mechanisms confer advantages through reduced oxygen dependence or full oxygen independence. Accordingly, future efforts should prioritize the development of Type I PSs and oxygen‐independent PACT‐active Pt complexes to overcome hypoxia‐associated resistance. More broadly, overcoming tumor hypoxia will likely also require multimodal strategies that integrate PDT or PACT with immunotherapy, radiotherapy, or photothermal therapy to counteract tumor heterogeneity and resistance. Sustained progress in molecular engineering, multimodal combinatorial approaches, and refined spatiotemporal control will be essential to realize the full therapeutic potential of targeted and Pt‐based PDT and PACT agents as cornerstone oncological therapies.

**TABLE 4:** Summary of PSs and photoactivated agents: Photophysical and photochemical properties, ROS mechanisms, and hypoxia relevance.

PS/photoactive agent category	Representative complex	Photophysical properties	Photochemical properties	ROS mechanism	Relevance to hypoxic tumor environments	Key advantages	Ref
*Diazido ligands*	**2** and **6**	Visible absorption	Photoreduction; concomitant ligand release (N_3_•/OH• radicals)	PACT	Highly relevant to hypoxia	O_2_‐independent Pt(II) release + radical species; excellent for hypoxic tumors	[52, 64]
*Porphyrins*	**4**, **5**, **15**, and **16**	Visible absorption	Rapid ^1^O_2_ generation; exceptional phototoxic indices	Type II	Limited in severe hypoxia	Potent PDT with low dark toxicity	[55, 56, 90, 91]
*AIE PS*	**8**	Visible absorption	Light‐induced ROS + Pt(IV) reduction to Pt(II) for synergistic therapy	Type II + PACT	Advantageous in hypoxia via the O_2_‐independent PACT pathway	Overcome aggregation‐caused quenching	[66]
*BODIPY derivatives*	**9, 11**, **18–22**, **28–30**, **32**, **34, 36**, and **37**	Green/red/NIR absorption (500–715 nm)	Rapid ^1^O_2_ generation; ligand dissociation upon irradiation of Pt(IV) prodrugs	Type II; Type I in specific designs	Moderate hypoxia relevance	Red/NIR light improves penetration; retains efficacy in hypoxic/resistant tumors	[70, 72, 104, 105, 106, 107, 108, 130, 133, 134, 139, 151, 154, 155]
*Coumarin derivatives*	**12** and **38**	Visible (**12**) or NIR (**38**) absorption.	Photoreduction, water‐oxidizing (**12**); NIR direct biomolecule photooxidation (**38**)	PACT	Highly favorable in hypoxia	O_2_‐independent mechanisms (water‐oxidizing or direct photooxidation); effective in hypoxic tumors; ER/nucleolus targeting enables unique non‐DNA damage pathways	[86, 157]
*Triphenylamine (TPA) derivatives*	**13** and **14**	Visible absorption	Efficient ROS production; DNA binding/interaction in the nucleus	Type II	Limited in severe hypoxia	Nucleus localization and DNA damage response provide high potency; potent for deep‐tissue two‐photon PDT	[87, 88]
*Heptamethine‐cyanines*	**23**	NIR absorption	Efficient ^1^O_2_ generation; Pt–O bond scission	Type II + PACT	Advantageous in hypoxia via the O_2_‐independent PACT pathway	Mitochondria‐targeting and NIR tissue penetration, overcoming hypoxia‐mediated PDT failure while maintaining imaging‐guided precision	[110]
*Rhodamine B (RhB)*	**25**	Visible absorption	Intramolecular photoswitch dramatically enhances the photoactivation rate	PACT	Highly favorable in hypoxia	Activation via O_2_‐independent, mitochondria targeting + efficient photoswitching overcomes activation barriers in cells	[115]
*Ru‐based derivatives*	**26, 27**	Visible absorption	Rapid cellular ROS production in Mt; efficient photodamage of mtDNA	Type II (**26**) or mixed (Type I/II; **27**)	Limited to good in hypoxia condition	Effective against resistant models via mitochondrial dysfunction	[117, 118]

Beyond these translational barriers, achieving an optimal balance between the intrinsic chemotherapeutic cytotoxicity of the Pt center and photodynamic activity is essential for achieving truly synergistic dual‐action modalities. Under normoxic conditions, excessive ROS generation can produce strongly PDT‐dominant phenotypes that obscure Pt contributions. Establishing this balance is therefore critical for enabling synergistic, oxygen‐independent multimodal cell death capable of overcoming both hypoxia‐imposed limitations and classical Pt resistance. To attain reliable dual‐action synergy, three interconnected strategies warrant systematic pursuit. First, structural optimization can be leveraged to elevate the local Pt concentration within target cells. One promising approach is the development of photoactivatable di‐ or even multi‐nuclear Pt complexes whose photodecomposition releases two or multiple mononuclear Pt(II) species alongside ROS generation [134, 199, 200], thereby intrinsically strengthening the Pt contribution upon irradiation. Second, rational axial ligand engineering in Pt(IV) prodrugs is needed to optimize dark stability and photoactivation kinetics. For instance, replacing labile carboxylate/hydroxido ligands with alkoxido or sterically tuned groups in carboplatin‐ or oxaliplatin‐based scaffolds, combined with optimized PS attachment, can prevent premature reduction, diminish dark cytotoxicity, and maintain efficient light‐triggered activation. This strategy enables the Pt(II) chemotherapeutic component to work in concert with PDT rather than being compromised by instability or masked by dominant ROS pathways [16, 155, 201, 202]. Third, iterative mechanistic validation coupled with feedback‐driven redesign is indispensable. During lead optimization, orthogonal quantification of Pt contributions (DNA adducts and protein platination) versus PDT effects (ROS generation, lipid peroxidation, O_2_ dependence) allows precise adjustment when assessed in hypoxia models, Pt‐resistant cell lines, radical scavenger conditions, and DNA repair‐deficient systems. Where PDT activity prevails, whether because lesions are readily repaired or because activity declines under low‐oxygen conditions, further ligand modification or incorporation of PACT motifs may be warranted. Conversely, when Pt‐mediated effects outweigh PDT activity, enhancing PS potency should become the priority. Collectively, these strategies offer a pathway to evolve photoactivatable Pt complexes from occasional “PDT‐only” performers into consistently effective synergistic multimodal therapeutics. Such advances should move the field closer to clinically practical options for tackling hypoxic tumors and Pt‐resistant cancers.

## Author Contributions

**Shu Chen**: conceptualization, writing–original draft, writing–review and editing. **Qiyuan Zhou**: writing–review and editing, writing–original draft. **Jiaqian Xu**: writing–review and editing, writing–original draft. **Guangyu Zhu**: conceptualization, funding acquisition, writing–review and editing, project administration, supervision, and resources.

## Conflicts of Interest

The authors declare no conflicts of interest.

## Data Availability

Data sharing is not applicable to this article as no new data were created or analyzed in this review.
